# Fate and health implications of the radioactive ultrafine aerosols in the air

**DOI:** 10.1038/s41598-025-10923-0

**Published:** 2025-07-09

**Authors:** Magdalena Długosz-Lisiecka, Myriam Lopes, Alexandra Monteiro, Joana Ferreira

**Affiliations:** 1 Faculty of Chemistry, Institute of Applied Radiation Chemistry, Lodz University of Technology, Wróblewskiego 15, Lodz, 90-924 Poland; 2 CESAM, Department of Environment and Planning, University of Aveiro, Campus Universitário de Santiago, Aveiro, 3810-193 Portugal

**Keywords:** Ultrafine particles, Aerosols, Radioactive isotopes, Radiological protection, Nuclear accident, Release of radionuclides emergency responds, Ecology, Chemistry

## Abstract

Fine and ultra fine particles with attached radioactive ions, often highly toxic and carcinogenic, can cause serious acute and chronic health issues. Due to environmental significance of nanoparticles associated with radioactive isotopes of natural or artificial origin and the worldwide health risk they pose, this article presents how they are transported into the troposphere. This study evaluates the public health impact of radionuclides released during radiological accidents, focusing on inhalation doses in contaminated regions from three cases: Chernobyl (1986), Fukushima (2011), and ruthenium-103/106 emissions (2017). It highlights the critical role of aerosol particle size and atmospheric residence time in health risk assessment and crisis decision-making. This information is crucial in the context of crisis management, evacuation planning, and future intervention actions, for radiation protection of the public. Knowledge about the fate of radionuclides, their concentration, and the dynamics of their washout from the atmosphere is particularly useful during rescue operations for responders working in the direct exposure region.

## Introduction

The aerosol size distribution is one of the most important parameters in modelling of particles dispersion and determination of the impact on human health, especially in case of internal contamination. Airborne particulate matter originates from various sources, with different formation mechanisms and chemical properties, and serves as a transport medium for radionuclides that are primarily absorbed on their surfaces^1–3^.

Suspended particles can be divided based on their size into: PM 10 dust—particle diameter is below 10 μm, PM 2.5 dust—particle diameter below 2.5 μm, submicron PM1 dust—diameter below 1.0 μm, and ultrafine PM 0.1 dust—particle diameter below 0.1 μm. The size of aerosols determines where they will penetrate the respiratory system during inhalation. Ultrafine, fine, and coarse particles impact the human body differently^4,5^. Particles with a diameter above 10 μm are trapped in the nose and throat, those with a diameter of 2.5–10 μm in the trachea or bronchi, while those below 2.5 μm penetrate into the lung alveoli. For mouth breathing, with an air flow rate of 1.8 m^3^/h, on average, 20% of particles with an aerodynamic diameter of 5 μm and about 70% with a diameter of 10 μm deposit before the larynx^6^. Nanoparticles can enter blood vessels and interact with the entire human body, impacting it significantly and generating internal exposure. Radioactive internal contaminations can cause acute and chronic health problems such as respiratory problems, acute bronchitis, heart problems, lung cancer, aggravation of preexisting heart and lung disease, and asthma. For an organism exposed to internal radiation, the type of radiation and its energy are of great importance. Therefore, each radionuclide is assigned with a different activity-to-dose conversion factor^7^. As a result of internal contamination, there is physical decay of the radioactive material within the body, burdening tissues and organs, but at the same time, there is physiological transport of the absorbed substance containing the radionuclide. In such cases, the magnitude of the acquired radiation dose depends not only on the type of ionizing radiation and its energy but also on the chemical compound in which the radionuclide is contained, the route of absorption, and the effective residence time. Radionuclides attached to aerosols can cause internal contamination, particularly during radiological accidents. However, they can also be used as tracers to identify particle sources introduced into the air and serve as isotopic clocks for measuring aerosol residence time. The residence time of aerosols defines the duration that aerosol particles remain suspended in the atmosphere before being removed or deposited onto surface layer of the ground^7,8^. The aerosol residence time in the air can vary significantly depending on the type of aerosol, diameter, atmospheric conditions especially with wet and dry washing out processes. Fine or ultrafine particles may be dispersed, removed, or decomposed by various atmospheric processes such as rainfall. Generally, fine aerosols can remain in the troposphere for a longer period from several days up to months, while larger particles may settle faster, even within a few hours^4,9^.

The fine nanoparticles with size lower than 1 μm (PM1) can be suspended in the atmosphere for a long time, allowing them to be transported over long distances and potentially affecting air quality in different regions. These particles are mainly generated by anthropogenic emissions through biomass and fossil fuel combustion processes^10–12^. Up to 95% of detected particles are < 100 nm and the weight% of submicron particles can be as high as 70–90% for industrial processes^13^ or energy production especially by biomass combustion^10,12^. The most volatile and toxic elements to living organisms especially at combustion temperatures—can enter the atmosphere not only through accidental releases but also as a result of industrial activities, particularly those involving high-temperature and high-pressure processes^14–16^.

Suspended dust composed of fine particle matter found in the air is classified by the International Agency for Research on Cancer as a carcinogen for humans. Over the past 35 years, the number of cancer cases has tripled (National Cancer Registry)^17^. According to the EEA report (European Environment Agency)^16^, it is estimated that about 467 000 premature deaths in 41 European countries in 2013 can be attributed to air pollution with PM 2.5 dust. There is an association between PM10 and non-fasting blood triglycerides, found by Gaio et al.^16^, and between PM10 and cardiovascular disease and decreased lung function (asthma)^10^.

In case of an radiological accident, released radionuclides are getting attached to the suspended particles, especially to these with the smaller nanometres size. In practice, the finest particles with low aerodynamic diameter are likely to be attached by isotopic ions, like ^137^Cs⁺,^131^I⁻ or ^131^I⁺, ^90^Sr²⁺. In this way isotopic contaminations attached to particles create highly mobile, radioactive aerosols^8,18^. In case of an accidental emission of radionuclides, as a result of nuclear power plant failure or other processes like furnace of isotopic material, or even nuclear weapons tests, accidents at nuclear facilities, or leaching from radioactive waste disposal facilities, volatile radionuclides start to spread freely to the environment. In some cases, the process can occur rapidly due to immediate conditions like fire or more slowly as a result of the gradual washing out of radionuclides in stages^19,20^. If the radionuclide amount injected into the atmosphere is very large and the level of isotopic contaminations attached to the submicrometric particles is also high, this remains dangerous for people exposed to this contaminated air, especially for a long time period^21^. Particles with a diameter < 0.5 μm may originate from a diffusion mechanism based on Brownian motion and diffusion as well as condensation occurring due to adiabatic expansion of exhaust gases associated with changes in their velocity and cooling. A high degree of mobility of artificial radionuclide is a result of the chemical properties of its ionic forms. The fate in the environment and the toxicity effects of radionuclides depend on the element’s chemical form^21^.

The behavior and distribution of radioactive isotopes following a nuclear explosion or radiological event are strongly influenced by the physical characteristics of aerosol particles in the atmosphere. Even 76% of the lead isotopes ^214^Pb-activity and 67% of the ^212^Pb-activity were found in the 0.08 to 1.4 μm size range of particles^22–24^. The more volatile fission products and those with volatile parents are distributed in the smaller particles. If explosion of the bomb occurs in the troposphere without contact with the ground or in case of a high-temperature process (e.g., fire), condensed aerosol particles are very small, with 90% of the activity in particles less than 0.3 μm in diameter^22^.

The Chernobyl (Chornobyl) accident in 1986, the Fukushima Daiichi nuclear power plant accident in 2011, and the unexplained atmospheric release of radioactive ruthenium isotopes (¹⁰³Ru and ¹⁰⁶Ru) in 2017 caused extensive air contamination that spread beyond national borders^25^. The 2017 ruthenium release, detected across Europe, was traced to a likely incident involving nuclear fuel reprocessing, although no country officially acknowledged responsibility for it. Following these accidents, emergency monitoring of radionuclide concentrations in the air was carried out to estimate internal dose for population and track the long-term fate of radioactive aerosols. During Chernobyl or Fukushima accidents, the release of radioactive material was multistage and consisted of gases, aerosols, and larger fragmented fuel^26^. Noble gases such as krypton, xenon, and partly iodine (50 to 60%), escaped dynamically without any chemical reaction or physical change of the gaseous phase. Volatile elements such as cesium and tellurium, attached to aerosols, were transported in the air with an activity median aerodynamic diameter (AMAD) ranging from 0.3 to 1.5 μm and with an AMAD of 10 μm, change from 0.25 to 0.71 μm for ^137^Cs, from 0.17 to 0.69 μm for ^134^Cs, and between 0.30 and 0.53 μm for ^131^I^18^. Non-volatile radionuclides such as ^95^Zr, ^95^Nb, ^140^La, and ^144^Ce were rather correlated with larger particles with AMAD superior to 10 μm.

The distribution of particles with different sizes resulting from accidental release is always non-homogenic. Fine particles can remain suspended in the atmosphere for longer periods and be transported over greater distances than large particles, potentially posing widespread health risks. [27]. Therefore, the radioactive nature of particles should be taken into account when assessing public health impacts and proper radiation protection should be provided to inhabitants of contaminated regions.

In previous studies, the fate and size distribution of radioactive particles released from different accidental events have been analyzed^8,12,28^. Due to various size distribution of radioactive particles, their fate in the air, residence time, and deposition effects in human respiratory tract system changed significantly. These parameters should be taken into account together with radiotoxicity, physical and biological half-life, or concentrations of radioactive isotope in the air, when modelling exposure and dose estimation^8,12,28^. The radionuclide size distribution can change due to the number and origin of natural particles suspended in the air, the pathway of radioactive plume, and the change in emission intensity during the accidental emission. Most of the radioactive aerosols after nuclear accidents can be carried on particles with a diameter of less than 2 μm^4^. Inhalation of air containing radionuclides with elevated activity accumulation on nanoparticles can lead to an increase in radiation dose; therefore, this methodology helps in the decision-making process after a radiation disaster^20^.

Different radioactive fractions of aerosols will cause different health effects on the human body upon inhalation. Considering all these parameters, it is important not to treat isotopic contamination in the air as a single, often simplified model. Experience gained and lessons learned from managing past incidents highlight the capability gaps in knowledge, technologies, and operational implementation. Therefore, it is crucial to make scientists aware of the range of risks associated with the distribution of isotopic contamination in aerosols in the air. Therefore, scientists cannot apply the same approach to assess the effects on human health in each case.

But the question is how long isotopic pollutions stay in the atmosphere and generate hazard for human organisms. How does the size distribution of radioactive aerosol particles released during accidental emissions influence their atmospheric behavior and how can incorporating these variables improve the accuracy of exposure assessment and radiation protection strategies? What are the impacts of ultrafine radioactive aerosol particles on human health compared to larger particles following nuclear accidents, and how can understanding their differing atmospheric residence times and biological behaviors inform more effective public health responses and dose estimation models? The purpose of this study is to estimate exposure to isotopic contamination as accurately as possible, which is particularly relevant for modelling dispersion and exposure estimation studies. For that, three selected large-scale radiation events of different nature (Chernobyl 1986, Fukushima 2011, and the latest event covering practically the entire Europe with its distribution, i.e., the emission of ^103,106^Ru) were selected to assess the actual participation of isotopic pollutants adsorbed on the surface of fine and very fine particulate matter in the radiological exposure of individuals.

## Materials and methods

To assess internal exposure to radioactive aerosols, this study employed a multidisciplinary approach that incorporates aerosol size distribution, radionuclide-specific parameters, and effective exposure modeling, aligned with guidance from the International Commission on Radiological Protection (ICRP), particularly Publication 59, 66, 71, and 78^29–33^. This framework enables improved estimation of inhalation doses based on particle deposition patterns and biokinetic behavior. The Activity Median Aerodynamic Diameter (AMAD) was determined for dominant isotopic accumulations in aerosols using published measurements from monitoring stations following the Chernobyl (1986), Fukushima (2011), and ruthenium-103/106 (2017) events. This step ensures that the size distribution of radionuclide-laden aerosols accurately reflects deposition within various regions of the respiratory tract, a key factor in internal dosimetry according to ICRP Publications^29–33^.

The aerosol residence time T_R_ was calculated based on the difference between the measured AMAD of radioactive radionuclides concentration on aerosols surface and the diameter of Aitken nuclei (AMDA) equal to 0.015 μm by the mean growth rate (MGR) of 0.004–0.005 μm/h (assumed 0.0045 μm/h) according to equation used previously^9^.

Time of real human exposition T_ef_ as a function of aerosol residence time, physical and its biological half-time, are important in estimation of internal exposure. In this approach, activity median aerodynamic diameter appropriately represents real size distribution of radionuclides attached to the particles, suspended in the open air and inhaled to the terminal bronchus, alveolar duct, or even deeply into the alveolus, and then into the blood vessels, causing contamination of the whole human body.

The effective impact time T_ef_ (Eq. 1) of radionuclides on the human body, in relation to the presence of isotopic contaminations in the air, after single injection event, will depend on the aerosol’s residence time in the air (including size distribution), the half-life, and the time of biological elimination from the body (including the chemical form of ions).

1 $$\frac{1}{{T}_{ef}}=\frac{1}{{T}_{1/2}}+\frac{1}{{T}_{biol}}+\frac{1}{{T}_{R}}$$

T_biol_ - biological half-life of a radioactive compound that has been removed from the biological organism.

T_1/2_ - half-life of radionuclides.

T_R_ – aerosols residence time calculated on the base of AMAD.

The classic approach (Eq. 2) to calculating internal exposure E associated with the inhalation of aerosols containing radioactive isotopes on their surface involves multiplying the absorbed amount of the radionuclide J [Bq] by the dose conversion factor DCF [Sv/Bq] for inhalation.

2 $$E=\sum J*DCF [SV]$$

However, this work adopts an improved approach that estimates the amount of the radionuclide J [Bq]. Previous studies^9,18^ described the typical particle sizes but did not combine these factors into a unified effective exposure time model.

A new approach (Eq. 3) to assess the absorbed amount of the radionuclide J considers the residence time of aerosols, breathing rate (medium type M), and the concentration of the nuclide in the atmosphere:

3 $$\text{J}=\text{C}\cdot {\text{T}}_{ef} \cdot \nu$$

Where:

C – radioactive isotope concentration in the atmosphere [Bq/m^3^],

T_ef_ – effective impact time T_ef_ of radionuclide on the human body, in relation to the presence of contaminations in the air [h],

ν – respiratory rate, dynamic [m^3^/h], in this approach equal to 1.2 [m^3^/h].

This modified equation better reflects the inhaled dose over time under changing environmental conditions and exposure durations. This new approach has been applied to the estimation of the inhalation dose in three cases of radiological accidents: Chernobyl, Fukushima, and ruthenium release, and the results are shown in the next section.

The method was applied to simulate three radiological events:

- Chernobyl (1986): Analysis of volatile and non-volatile radionuclides (e.g., ^137^Cs, ^131^I, ^90^Sr).
- Fukushima (2011): Size distribution data for ^134,137^Cs and ^131^I isotopes.
- Ruthenium-103/106 ^103,106^Ru release (2017): Modeled using European monitoring data and dose coefficients.

Where possible, calculated dose estimates were cross-referenced with reported public exposures from NEA^33^, IRSN^34^, and Rosgidromet^35^. All internal exposure modeling adhered to ICRP’s biokinetic and dosimetric models for public exposure, specifically those outlined in Publications 59, 66, 71, and 78^29–33^.

## Results

This study introduces a new method for estimating inhalation doses that incorporates aerosol residence time T_R_ and effective impact time T_ef_, improving the accuracy of exposure assessment during radiological events.

**Fig. 1:**
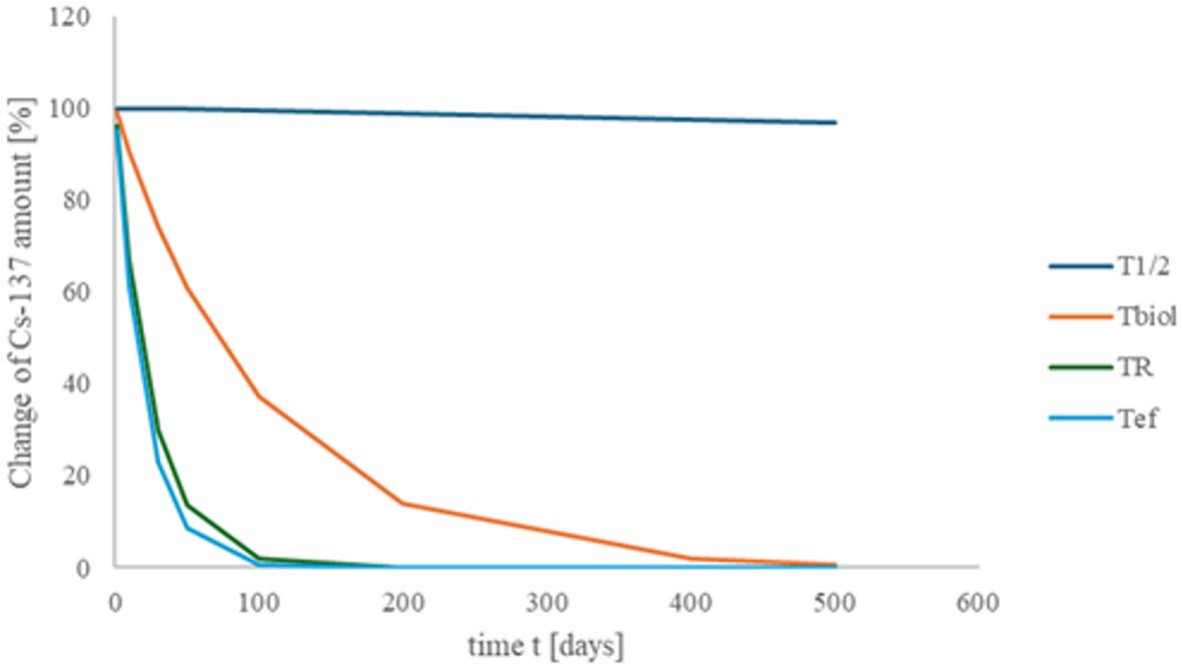
The dynamic of ^137^Cs decrease correlated with radioactive decay T_1/2_, biological half-life T_biol_, aerosols residence time T_R_, and the dynamic of effective interaction with human body T_ef_.

Figure 1 shows the dynamic of ^137^Cs reduction, caused by several processes like radioactive decay T_1/2_, biological half-life T_biol_, aerosols residence time T_R,_ and the dynamic of effective interaction with human body T_ef_. Using the proposed approach, the effective interaction time (Tef) of radionuclides with the human body was calculated, based on the result from integrating aerosol atmospheric residence time, radionuclide decay, and biological elimination rates. For ^137^Cs radionuclide, effective time of interaction with human body seems to be quite short and equal to 14 days (see Table 1). In case of ^106^Ru isotope, time of effective interaction was shorter and equal to 8.4 days. ^239^Pu isotope has a long time of radioactive decay T_1/2_, biological half-life T_biol_, but the effective impact time T_ef_ has been determined by aerosols residence time and is equal to about one month.

**Table 1:** List of artificial isotopes and their features.

isotope	T_1/2;_ T_biol;_ T_*R*;_ T_ef_	AMAD	DCF dose conversion factor Inhalation for public in age > 17y based on ICRP (Sv/Bq)
^90^Sr	29.1 y;18,000 d; 28.5 d; 14.1d	3.1–13 μm (Garger et al., 2006)	3.6 10^−8^ (ICRP 1993, 1997)
^106^Ru	1.01 a;1000 days (in 20%), 8.29 d; 8.40 d	0.39–0.91 μm (Mala, et al., 2013)	2.8 10^−8^ (ICRP 1992, 1993, 1995, 1997)
^137^Cs	30 y; 70 d;17.3; 14.1d	0.25 TO 0.71 μm (Masson et al. 2013) 0.53–1.93 (Kaneyasu et al., 2017)	9.7 10^−9^ (ICRP 1992, 1993, 1997)
^134^Cs	2.06 years, 70 days;7.9d;7.1d	from 0.17 to 0.69 μm (Masson et al. 2013) 0.54 and 0.87 μm (Kaneyasu et al. 2017)	9.1 10^−9^ (ICRP 1992, 1993, 1997)
^131^I	8.04 days, 138 days;6.3d; 3.4d	0.30 to 0.53 μm (Masson et al.???	2.4 10^−9^ (ICRP 1992, 1993, 1997)
^239^Pu	2.41 10^4^ a 50 years in bones (Gückel et al. 2017) 32.3 d, 32.3 d	3.5–11 μm (Garger et al. 2006)	5.0 10^−5^ (ICRP 1993, 1997)
^240^Pu	6.54 10^3^ a ;50 y in bones (Gückel et al. 2017) 32.3 d 32.3 d	3.5–11 μm (Garger et al. 2006)	5.0 10^−5^ (ICRP 1993, 1997)
^241^Am	432.2 y 50 years in bones Gückel et al. 2017 13.75d 13.75d	1.5 μm (Garger et al. 2006)	4.2 10^−5^ (ICRP 1993, 1997)

Note: Assumed lung absorption type M (medium).

Simulation programs for radiological events do not consider the aerosol residence time or the effective exposure time for the general population, emergency responders, or cleanup workers. The simulation of radioactive contamination distribution reveals that the behavior of radionuclides in the atmosphere is significantly influenced by their physical and chemical properties, as well as environmental and release conditions. A key challenge lies in determining whether the contamination exists in the solid phase, represented by particles of a specific size (e.g., 1 μm), or in the gaseous phase. Our findings demonstrate that radionuclides exhibit varying chemical forms, release histories, and dynamics of accumulation on aerosol surfaces, which collectively determine their atmospheric fate and deposition processes.

The study highlights that radionuclides’ behaviors are strongly correlated with properties such as vapor pressure, solubility, chemical reactivity, and affinity to particulate matter. Additionally, the source term and release conditions, including release height, duration, and magnitude, significantly influence isotopic dispersion in the atmosphere.

Once radionuclides attach to particles, their fate is governed by environmental factors, such as temperature, humidity, atmospheric pressure, and wind speed. For example, higher temperatures and lower humidity were found to favor vapor-phase transport, while cooler temperatures and higher humidity promoted particulate deposition. Atmospheric dynamics are also critical in dispersion, dilution, and eventual deposition of radionuclides onto surfaces [36].

The research further indicates that particle suspension time in the atmosphere correlates with particle growth. Larger particles tend to settle more rapidly due to gravitational forces, whereas smaller particles remain suspended for longer periods, potentially facilitating isotopic transport in the vapor phase or as nanoparticles. This finding underscores the importance of particle size in determining deposition dynamics in the surface layer.

The behaviors and properties of radionuclides in the atmosphere and their interactions with environmental conditions, as well as the size and understanding the deposition dynamics of different radionuclides based on their physical and chemical properties, will be essential for proper modelling how radioactive particles disperse in the atmosphere considering their phase (solid or gaseous). This includes analyzing how particles of different sizes settle and accumulate on surfaces under varying environmental conditions and what is the potential impact of radioactive contamination on human health. Early warning monitoring networks and national and international response strategies have a significant impact on responding to incidents by predicting areas of higher deposition and potential hot spots.

Understanding all these factors is crucial for accurately predicting the behavior of radionuclides in the environment following events such as nuclear accidents or atmospheric releases. During a nuclear event several characteristic radionuclides can be freely released.

Even up to 60% of iodine isotopes escapes in the gaseous form, the remaining 40% of the iodine portion prefers to attach to particles with aerosol diameter between 0.30 and 0.53 μm and Sr isotopes prefer 3.1–13 μm^18^. Velocity, aerosol residence time, and possible scale of dispersion in open air are dramatically different for both regions of particle sizes and phases. As a result, the concentration of radionuclides in the air [Bq/m^3^] in various distances from the source point and the surface contamination of the ground [Bq/m^2^] are different.

**Fig. 2:**
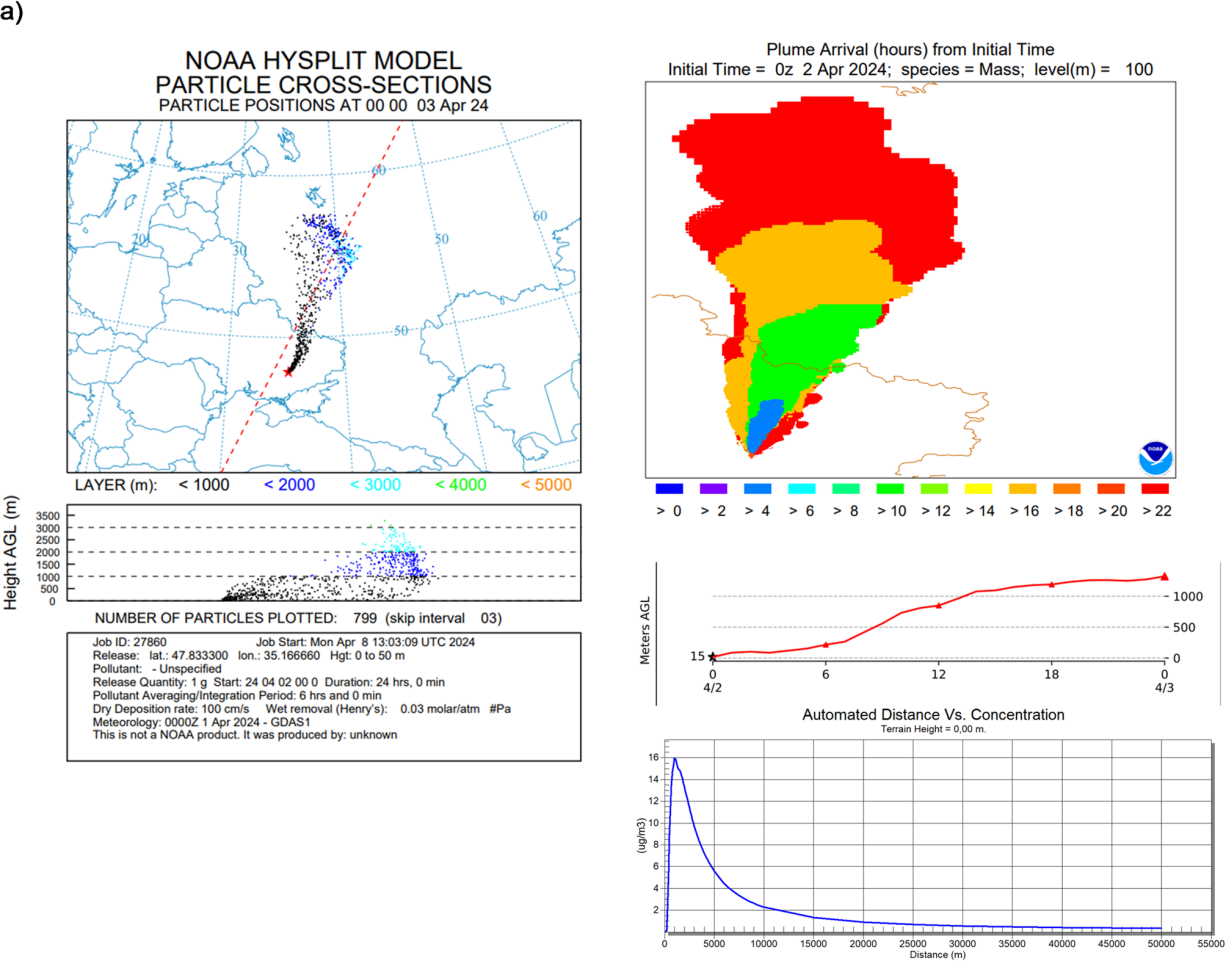

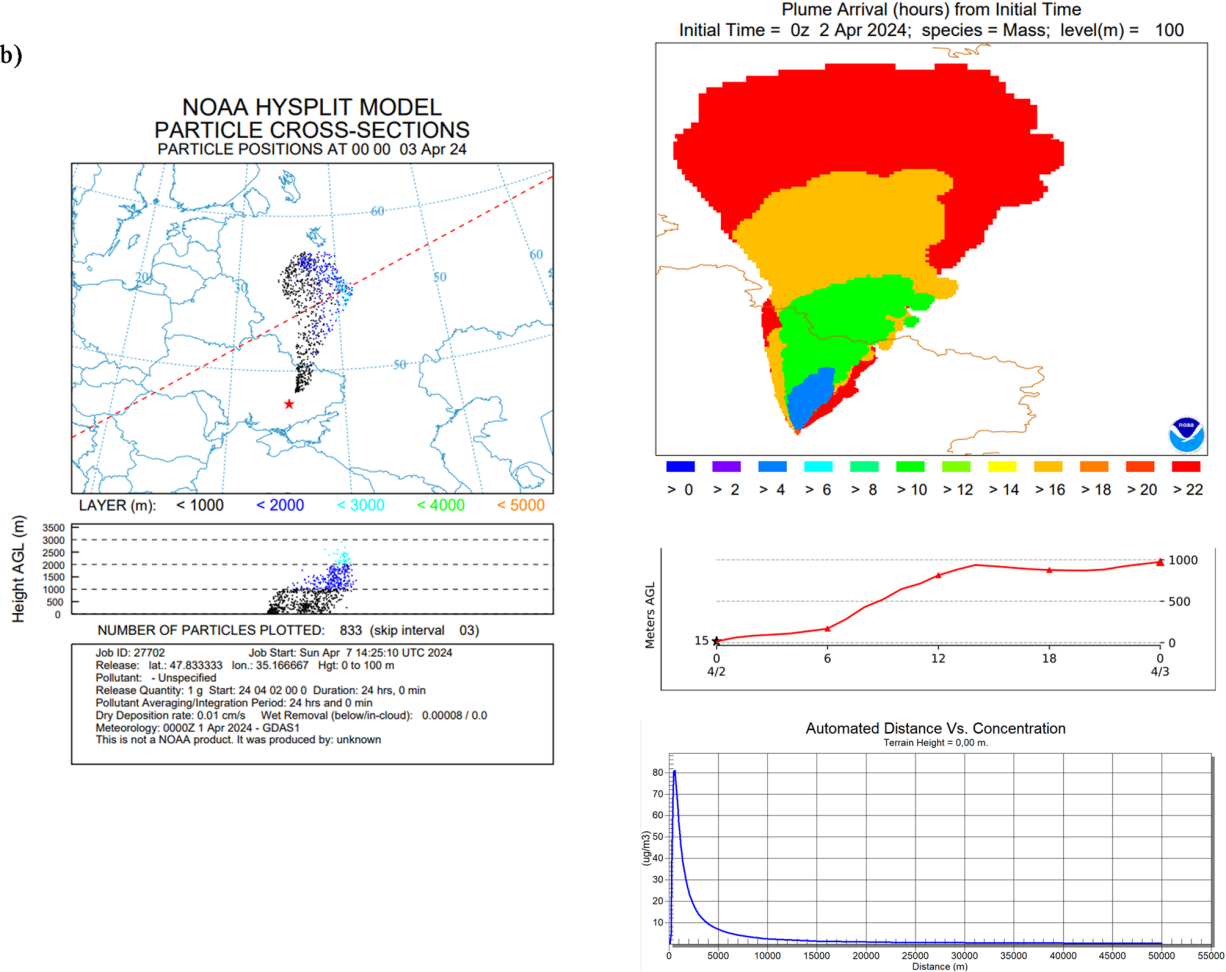

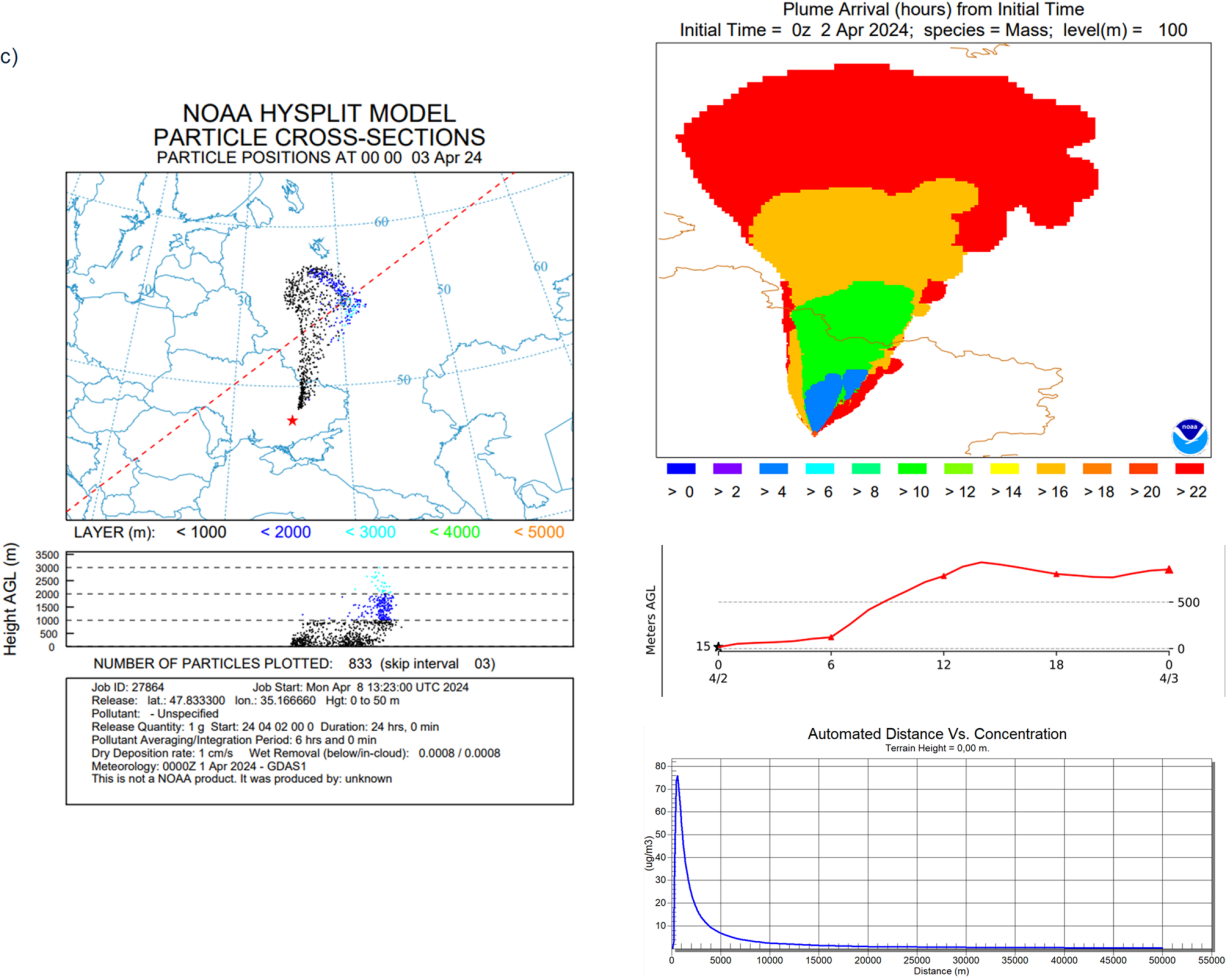
Simulation of the three case studies analyzed for Chernobyl: (**a**) with radionuclides in the gaseous form, (**b**) aerosols with a diameter of 0.5 μm and (**c**) aerosols with 5 μm diameter form (HYSPLIT dispersion model provided by NOAA Air Resources Laboratory, version 5.2.1, with applied GDAS meteorological data version, https://www.ready.noaa.gov).

In Hysplit velocity, settling of particles with size 0.5 μm is equal to 0.01 cm/s and particles with size equal to 5 μm, equal to 1 cm/s, were applied (based on Farmer^37^, Alvarado^38^ (Aerosol Particles with a density of 1 g/cm^3^ in the air at 20 ℃)) (Fig. 2.). It means that during one single day particles with size equal to 0.5 μm will move for about 8.65 m as a result of Brownian diffusion, when particles with size 5 μm will move for about 860 m in dry conditions by gravitational settling.

There is a huge difference between deposition processes dynamics for particles with various sizes; therefore, there is no single modelling approach^39–41^ and there are several issues with the internal dose calculation in case of the presence radioactive contaminations in the atmosphere (Fig. 2).

In these studies, various forms of isotopic contaminations—gaseous and suspended particles with two different sizes—have been taken into account. Figure 2 (panels a, b,c) shows Chernobyl accident with three versions, with all radionuclides emitted in the same portion of the activity but in different forms. Panel a) represents emission of isotopes in the gaseous form. Panel b and c) represent particles emission of isotopic contaminations with size 0.5 μm (velocity equal to 0.01 cm/s) and particles with size equal to 5 μm (velocity equal to 1 cm/s). Simulation results demonstrate that particles smaller than 0.5 μm stay suspended longer, leading to higher potential inhalation doses compared to larger particles (e.g., 5 μm), even if the initial activity was the same. Literature [5,22,39–41] describes that smaller aerosols have longer atmospheric residence times and higher penetration into lung alveoli but did not systematically connect it to real exposure duration modeling after major accidents.

In this study, emission conditions were not taken into account, only the physical form of the radionuclides. To normalize the calculations, all emission simulations considered a total emission of 1 g of gases or particulates. In the case of radionuclides emitted in the gaseous form, they reached higher parts of the atmosphere, > 3000 m above ground level (AGL). For gas emissions, the maximum concentration achieved was 16 µg/m³, reached just within the immediate area of the emission point, up to 2000 m. In the case of particulate emissions of varying sizes, the pollutants are more condensed and accumulate at lower altitudes. Consequently, they are washed out of the atmosphere to the ground quicker. The highest concentration of isotopic pollutants can reach up to 80 µg/m³ and 78 µg/m^3^, respectively, for particles with 0.5 and 5 μm size.

In practice, this means that gaseous isotopic contamination quickly spreads through large volumes of air, but generates radioactive fallout in a slow, long-term, and large-scale manner. With this type of emission, radioactive fallout will show lower concentrations, but larger areas will be contaminated. In the case of particulate emissions with a diameter of 5 μm, the contamination will be strongly correlated with weather conditions and localized to specific areas of emission.

Table 2 contains literature results of release to the environment of various radionuclides (in PBq, TBq, or GBq) during Chernobyl (1986), Fukushima (2011), and release of ruthenium ^103,106^Ru (2017) accidents. These data have been used for estimation of maximum radionuclide concentration outside of zona region [Bq/m^3^]. Radionuclides concentration in the air allows for estimation of the maximum dose rate in the peak of exposition [mSv/h] (exposure for population living outside of zona region).

**Table 2:** Released isotopes during radiological accidents, radiotoxicity of emitted isotopes.

Case study disaster	Isotopes released during disaster	Total estimated activity emitted during accident	Estimated max. radionuclide concentration outside of zona region[Bq/m^3^]	Estimated max. dose rate in the peak of exposition [mSv/h]
Charnobyl ‘86 (Christodouleas et al. 2011;NEA Report 2002)	^131^I ^90^Sr ^137^Cs ^134^Cs ^239^Pu ^240^Pu	1760 PBq 10 PBq 85 PBq 54 PBq 0.030 PBq 0.042 PBq	3,200,000 18,182 154,545 85,455 24 33	3.69 0.79 1.80 0.93 1.42 1.93
Sum:				10.6
Fukushima ‘11 (Raport MEGJ 2011)	^131^I ^90^Sr ^137^Cs ^134^Cs ^239^Pu ^240^Pu	160 PBq 0.14 PBq 15 PBq 18 PBq 3.2 GBq 3.2 GBq	290,909 23.1 2479 2975 0.00053 0.00053	0.335 0.001 0.029 0.032 0.00003 0.00003
Sum:				0.398
Ruthenium isotope emission ^106^ Ru ’17 (between 25 September and 28 September, release not exceed 24 h) (Report IRSN 2018)	^106^Ru	100 to 300 TBq	0.150 (max measured value)	0.000005

Based on Hysplit modelling, the specific isotopes concentration in the air (Bq/m^3^) in various distance from the point of emission were calculated (Fig. 2., max values collected in Table 2).

Taking into account the effective impact time T_ef_, we calculated total doses for Chernobyl (Ukraina, outside the zona), Fukushima (Japan, outside), and Europe citizens (region with the highest fallout). In these studies, the max release of isotopes has been taken into account. Isotopes emitted during accidents have been applied to estimate isotopes concentrations in the atmosphere and dose rate for inhabitants of the contaminated regions.

The estimated inhalation doses received by the populations in the highest contaminated Chernobyl region, during the first month could reach 300 mSv, while in Fukushima expected maximum was equal to 12 mSv. These values are comparable with those presented in the NEA Report^33^. In case of ruthenium release, the Report of IRSN^34^ noticed that the range of concentrations varied from some tenths µBq/m^3^ to more than one hundred mBq/m^3^.

In this study, we obtained max concentration of ^106^Ru equal to 150 µBq/m^3^, and inhalation dose received by an adult person, exposed to ^106^Ru present in air equal to then 1 $\mu$ Sv taking into account 8.4 days of effective impact time T_ef_ of an isotope on the human body in the affected region. Rosgidromet^35^ report indicates very high depositions up to 340 Bq/m^2^ for Metlino (Russia) in a station close to Mayak and concentration of gross beta emitters 1570 µBq/m^3^ in Upper Dubrovo (Ural).

Bossew^19^ noticed that calculated doses received in Europe vary between less than 0.3 µSv to unobserved maxima up to 0.4 µSv. For a number of European cities, where ^106^Ru was detected, inhalation doses between about 1 nSv and 0.33 µSv could be estimated.

## Discussion & conclusions

Studying the fate and health effects of the radioactive ultrafine aerosols in the air is important because it may contribute to a better understanding of the fate of radionuclides in the atmosphere, their different distribution depending on the diameter of the aerosols, different impact on the kinetics of absorption in the human body, and, in practice, a different inhalation dose related to internal contamination. This study aimed to assess the impact of radioactive isotopes released into the atmosphere during radiological accidents on public health, estimating the inhalation doses received by the population in contaminated regions, based on three cases: Chernobyl (1986), Fukushima (2011), and the emission of ruthenium-103 and 106 (2017). Particular emphasis was placed on the significance of aerosol particle size and atmospheric residence time, which are crucial for evaluating health risks and making decisions in crisis situations.

The three analyzed case studies have shown that the highest inhalation doses are associated with aerosols with the smallest aerodynamic diameter—lower than 0.5 μm—related with the highest amount of radionuclides attached on the particles surface or in the gas phase. These results suggest that significant impact on human body can exist, by deep interaction and distribution of isotope in most morphological parts, only in case of presence of high concentrations of radionuclides attached on the particles, especially those with smaller nanometer sizes. If large amounts of radionuclides are injected into the atmosphere and attach to submicrometric particles, they pose a significant health risk, particularly through the inhalation of contaminated air over extended periods.

The high mobility of artificial radionuclides, the environmental fate, and toxicity effects of radionuclides are influenced by their chemical form. For instance, explosions or high-temperature processes produce small aerosol particles, leading to significant distribution of isotopic contamination. During the Chernobyl accident, the release of radioactive material was multistage, involving gases, aerosols, and larger fragments. Volatile elements like cesium Cs and tellurium Te attached to aerosols were transported with specific aerodynamic diameters. Non-volatile radionuclides correlated with larger particles. Therefore, the distribution of contaminations from accidental releases is non-homogeneous, with larger particles causing acute health hazards over larger regions. Accurate assessment of radioactive particle impact on public health requires considering particle size, radiotoxicity, and residence time in the air.

These results have confirmed that radioactive isotopes attached to the suspended particles have a significant impact on public health by synergy between radiotoxic feature of radionuclides and penetration ability of the particles. Fate of radionuclides suspended in the atmosphere as a result of an accidental situation needs to be controlled, and modelled to help in decision making and risk assessment. But significant contribution to public health results from aerosol residence time, chemical compounds, size of particles, and meteorologic parameters and washing out processes (wet or dry).

In this approach, a new methodology to estimate inhalation doses from radioactive aerosols by incorporating aerosol size distribution and aerosol residence time into the exposure model was proposed. The concept of effective impact time (Tef)—combining aerosol atmospheric residence time, radionuclide physical half-life, and biological half-life—was applied to better model the duration of internal exposure. Smaller ultrafine particles (< 0.5 μm), which have longer atmospheric residence times and deeper lung penetration, pose a significantly higher health risk during radiological accidents than larger particles or gaseous forms. Comparing three major radiological events (Chernobyl 1986, Fukushima 2011, and the 103/106Ru release 2017) using this new approach, highlighted the underestimated health risks associated with submicron and ultrafine radioactive aerosols.

## Data Availability

Data AvailabilityThe data are available: https://doi.org/10.34658/RDB.JJKQUF.

## References

[1] Kabdyrakova, A. M. et al. Distribution of artificial radionuclides in particle-size fractions of soil on fallout plumes of nuclear explosions. *J. Environ. Radioact*. **186**, 45–53. 10.1016/j.jenvrad.2017.09.022 (2018). 28985989 10.1016/j.jenvrad.2017.09.022

[2] Singleton, A. A. et al. Effects of grain size, mineralogy, and acid-extractable grain coatings on the distribution of the fallout radionuclides ^7^Be, ^10^Be, ^137^Cs, and ^210^Pb in river sediment. *Geochim. Cosmochim. Acta*. **197**, 71–86. 10.1016/j.gca.2016.10.007 (2017).

[3] Khalaf, H. N., Mostafa, M. Y. A., Vasyanovich, M. & Zhukovsky, M. Comparison of radioactive aerosol size distributions (Activity, number, mass, and surface area). *Appl. Radiat. Isot.* **145**, 95–100. 10.1016/j.apradiso.2018.12.022 (2019). 30590349 10.1016/j.apradiso.2018.12.022

[4] Marval, J. & Tronville, P. Ultrafine particles: A review about their health effects, presence, generation, and measurement in indoor environments. *Build. Environ.* **216**, 0360–1323. 10.1016/j.buildenv.2022.108992 (2022).

[5] Saya, A., Yoshikane, T., Chang, E. C., Yoshimura, K. & Oki, T. Precipitation redistribution method for regional simulations of radioactive material transport during the Fukushima Daiichi nuclear power plant accident. *J. Geophys. Res. Atmos.* **123**, 10248–10259. 10.1029/2018JD028531 (2018).

[6] Morawska, L., Keogh, D. U., Thomas, S. B. & Mengersen, K. Modality in ambient particle size distributions and its potential as a basis for developing air quality regulation. *Atmos. Environ.* **42**, 1617–1628. 10.1016/j.atmosenv.2007.09.076 (2008).

[7] Feng, X. et al. Combination of multiple isotopes and PMF model provide insights into the method optimization of PM_2.5_ source apportionment during haze episodes. *J. Geophys. Res. Atmos.* **129**, e2024JD041350. 10.1029/2024JD041350 (2024).

[8] Długosz-Lisiecka, M. & Bem, H. Aerosol residence times and changes in radioiodine-^131^I and radiocaesium-^137^Cs activity over central Poland after the Fukushima-Daiichi nuclear reactor accident. *J. Environ. Monit.* **14** (5), 1483–1489. 10.1039/c2em00014h (2012). 22481111 10.1039/c2em00014h

[9] Długosz-Lisiecka, M. Aerosol removal coefficients based on ^7^Be, ^210^Pb, and ^210^Po radionuclides in the urban atmosphere. *J. Atmos. Chem.* **78** (3), 209–218. 10.1007/s10874-021-09422-z (2021).

[10] Ali, M. U. et al. Pollution characteristics, mechanism of toxicity and health effects of the ultrafine particles in the indoor environment: current status and future perspectives. *Crit. Rev. Environ. Sci. Technol.* **52** (3), 436–473. 10.1080/10643389.2020.1831359 (2022).

[11] Długosz-Lisiecka, M. & Nowański, S. Factor regression as a tool to predict Be-7 concentration in the air. *Atmos. Environ.* **297**10.1016/j.atmosenv.2023.119612 (2023).

[12] Długosz-Lisiecka, M. The sources and fate of ^210^Po in the urban air: a review. *Environ. Int.* **94**, 325–330. 10.1016/j.envint.2016.06.002 (2016). 27295049 10.1016/j.envint.2016.06.002

[13] Riffault, V. et al. Fine and ultrafine particles in the vicinity of industrial activities: A review. *Crit. Rev. Environ. Sci. Technol.* **45**, 2305–2356. 10.1080/10643389.2015.1025636 (2015).

[14] Lopes, D. et al. An exploratory approach to estimate point emission sources. *Atmos. Environ.* **312**, 120026. 10.1016/j.atmosenv.2023.120026 (2023).

[15] Coelho, S., Ferreira, J., Carvalho, D. & Lopes, M. Health impact assessment of air pollution under a climate change scenario: methodology and case study application. *Sustainability*10.20944/preprints202209.0207.v1 (2022).

[16] EEA. Air quality in Europe — 2016 report, EN PDF: TH-AL-16-027-EN-N - ISBN: 978-92-9213-824, (2016). REPORT https://www.eea.europa.eu/en/analysis/publications/air-quality-in-europe-2016

[16] Gaio, V. et al. PM_10_ exposure interacts with abdominal obesity to increase blood triglycerides: a cross-sectional linkage study. *Eur. J. Public. Health*. **32** (2), 281–288. 10.1093/eurpub/ckab190 (2022). 34788428 10.1093/eurpub/ckab190 PMC9090274

[17] Religioni, U. Cancer incidence and mortality in Poland. *Clin. Epidemiol. Glob Health*. **8**, 329–334. 10.1016/j.cegh.2019.12.014 (2020).

[18] Masson, O. et al. F., Size distributions of airborne radionuclides from the Fukushima nuclear accident at several places in Europe. *Environ Sci Technol*. 1;47(19):10995 – 1003. (2013). 10.1021/es401973c. Epub 2013 Sep 18. PMID: 24001315. 10.1021/es401973c 24001315

[19] Bossew, P. et al. An episode of Ru-106 in air over europe, September–October 2017 – Geographical distribution of inhalation dose over Europe. *J. Environ. Radioact*. 205–206. 10.1016/j.jenvrad.2019.05.004 (2019). 79–92,ISSN 0265-931X. 10.1016/j.jenvrad.2019.05.004 31121424

[20] Długosz-Lisiecka, M. Public health decision making in the case of the use of a nuclear weapon. *Int. J. Environ. Res. Public. Health*. **19** (19), 12766. 10.3390/ijerph191912766 (2022). 36232066 10.3390/ijerph191912766 PMC9564949

[21] Taci, X. et al. Minutes to hours after a nuclear event: available radiation poisoning antidotes and practical considerations on possible urgent approaches. *Eur. J. Nucl. Med. Mol. Imaging*. **50** (12), 3498–3505. 10.1007/s00259-023-06305-1 (2023). PMID: 37367964; PMCID: PMC10547657. 37367964 10.1007/s00259-023-06305-1 PMC10547657

[22] Papastefanou, C. Radioactive aerosols. In: (ed Papastefanou, C.) Radioactivity in the Environment, Elsevier, 11–58. 10.1016/S1569-4860(07)12002-7 (2008).

[23] Długosz, M., Grabowski, P. & Bem, H. ^210^Pb and ^210^Po radionuclides in the urban air of lodz, Poland. *J. Radioanal Nucl. Chem.* **283**, 3, 719–725. 10.1007/s10967-009-0407-x (2010).

[24] Uddin, S., Fowler, S. W. & Behbehani, M. ^210^Po in the environment: reassessment of dose to humans. *Sustainability* **15**, 1674. 10.3390/su15021674 (2023).

[25] Tatsuno, T. et al. Effect of radioactive cesium-rich microparticles on radioactive cesium concentration and distribution coefficient in rivers flowing through the watersheds with different contaminated condition in Fukushima. *J. Environ. Manage.* **329**, 0301–4797. 10.1016/j.jenvman.2022.116983 (2023). 10.1016/j.jenvman.2022.116983 36565500

[26] Shirai, K. et al. Factors influencing acceptability of final disposal of incinerated Ash and decontaminated soil from tepco’s Fukushima Daiichi nuclear power plant accident. *J. Environ. Manage.* **345**, 0301–4797. 10.1016/j.jenvman.2023.118610 (2023). 10.1016/j.jenvman.2023.118610 37536131

[27] Pöllänen, R., Valkama, I. & Toivonen, H. Transport of radioactive particles from the Chernobyl accident. *Atmos. Environ.* **31**, 3575–3590. 10.1016/S1352-2310(97)00156-8 (1997).

[28] Długosz-Lisiecka, M. & Bem, H. Determination of the mean aerosol residence times in the atmosphere and additional ^210^Po input on the base of simultaneous determination of ^7^Be, ^22^Na, ^210^Pb, ^210^Bi and ^210^Po in urban air, *J. Radioanal. Nucl. Chem.* 293(1), 135–140, (2012). 10.1007/s10967-012-1690-5, b. 10.1007/s10967-012-1690-5 PMC4514680 26224924

[29] ICRP 59. The biological basis for dose limitation in the skin. ICRP publication 59. *Ann. ICRP* **22** (2),75-103, (1992). https://www.icrp.org/1812796

[30] ICRP 67. Age-dependent doses to members of the public from intake of Radionuclides - Part 2 ingestion dose coefficients. ICRP publication 67. *Ann. ICRP*. **23**, 3–4 (1993). https://www.icrp.org/7978694

[31] ICRP 71. Age-dependent doses to members of the public from intake of Radionuclides - Part 4 inhalation dose coefficients. ICRP publication 71. *Ann. ICRP*. **25**, 3–4 (1995). https://www.icrp.org/8735008

[32] ICRP 78, Individual Monitoring for Internal Exposure of Workers. ICRP Publication 78. *Ann. ICRP* **27** (3–4) https://www.icrp.org/ (1997). 9775348

[33] Report, N. E. A. Chernobyl: Assessment of Radiological and Health Impacts, Update of Chernobyl: Ten Years On. Nuclear Energy Agency, (2002). https://www.oecd-nea.org/jcms/pl_28256/

[34] Report, I. R. S. N. Report on the IRSN’s investigations following the widespread detection of ^106^Ru in Europe early October 2017. *IRSN Report* (2018).

[35] Rosgidromet БЮЛЛЕТЕНЬ о радиационной обстановке на территории России в сентябре/октябре/ноябре 2017 г. *Rosgidromet Report* (In Russian) (2017). http://egasmro.ru/ru/data/overal/refradsit/roshydromet

[36] Ashraf, M. A., Akib, S., Mohd Maah, J., Yusoff, I. & Balkhair, K. S. Cesium-137: Radio-Chemistry, fate, and transport, remediation, and future concerns. *Crit. Reviews Environ. Sci. Technol.* **44** (15), 1740–1793. 10.1080/10643389.2013.790753 (2014).

[37] Farmer, D. K., Boedicker, E. K. & DeBolt, H. M. Dry deposition of atmospheric aerosols: approaches, observations, and mechanisms. *Annu. Rev. Phys. Chem.* **72**, 375–397. 10.1146/annurev-physchem-090519-034936 (2021). 33472381 10.1146/annurev-physchem-090519-034936

[38] Alvarado, J. et al. Anthropogenic radionuclides in atmospheric air over Switzerland during the last few decades. *Nat. Commun.* **5**, 3030. 10.1038/ncomms4030 (2014). 24398434 10.1038/ncomms4030

[39] Długosz-Lisiecka, M. & Perka, D. Modelling of ^210^Pb and ^210^Po radionuclide emissions from local power plants in central Poland. *Environ. Sci. : Process. Impacts*. **22** (11), 2291–2297. 10.1039/d0em00141d (2020). 33112309 10.1039/d0em00141d

[40] Długosz-Lisiecka, M. & Bem, H. Seasonal fluctuation of activity size distribution of ^7^Be, ^210^Pb, and ^210^Po radionuclides in urban aerosols. *J. Aerosol Sci.* **144**10.1016/j.jaerosci.2020.105544 (2020).

